# Degree of Conversion and BisGMA, TEGDMA, UDMA Elution from Flowable Bulk Fill Composites

**DOI:** 10.3390/ijms17050732

**Published:** 2016-05-20

**Authors:** Edina Lempel, Zsuzsanna Czibulya, Bálint Kovács, József Szalma, Ákos Tóth, Sándor Kunsági-Máté, Zoltán Varga, Katalin Böddi

**Affiliations:** 1 Department of Restorative Dentistry and Periodontology, University of Pécs, 5 Dischka Gy Street, Pécs H-7621, Hungary; varga.zoltan@gmail.com; 2 Department of General and Physical Chemistry, University of Pécs, 6 Ifjúság Street, Pécs H-7624, Hungary, czibulya.zsuzsanna@gmail.com (Z.C.); balint621@gmail.com (B.K.); kunsagi@gamma.ttk.pte.hu (S.K.-M.); 3 János Szentágothai Research Center, University of Pécs, 20 Ifjúság Street, Pécs H-7624, Hungary; 4 Department of Oral and Maxillofacial Surgery, University of Pécs, 5 Dischka Gy Street, Pécs H-7621, Hungary; szalma.jozsef@pte.hu; 5 Faculty of Sciences, University of Pécs, 6 Ifjúság Street, Pécs H-7621, Hungary; totha@gamma.ttk.pte.hu; 6 Department of Biochemistry and Medical Chemistry, University of Pécs, 12 Szigeti Street, Pécs H-7624, Hungary; katalin.boddi@aok.pte.hu

**Keywords:** bulk-fill composite, degree of conversion, monomer elution, micro-Raman spectroscopy, HPLC

## Abstract

The degree of conversion (DC) and the released bisphenol A diglycidyl ether dimethacrylate (BisGMA), triethylene glycol dimethacrylate (TEGDMA) and urethane dimethacrylate (UDMA) monomers of bulk-fill composites compared to that of conventional flowable ones were assessed using micro-Raman spectroscopy and high performance liquid chromatography (HPLC). Four millimeter-thick samples were prepared from SureFil SDR Flow (SDR), X-tra Base (XB), Filtek Bulk Fill (FBF) and two and four millimeter samples from Filtek Ultimate Flow (FUF). They were measured with micro-Raman spectroscopy to determine the DC% of the top and the bottom surfaces. The amount of released monomers in 75% ethanol extraction media was measured with HPLC. The differences between the top and bottom DC% were significant for each material. The mean DC values were in the following order for the bottom surfaces: SDR_4mm_20s > FUF_2mm_20s > XB_4mm_20s > FBF_4mm_20s > XB_4mm_10s > FBF_4mm_10s > FUF_4mm_20s. The highest rate in the amount of released BisGMA and TEGDMA was found from the 4 mm-thick conventional flowable FUF. Among bulk-fills, FBF showed a twenty times higher amount of eluted UDMA and twice more BisGMA; meanwhile, SDR released a significantly higher amount of TEGDMA. SDR bulk-fill showed significantly higher DC%; meanwhile XB, FBF did not reach the same level DC, as that of the 2 mm-thick conventional composite at the bottom surface. Conventional flowable composites showed a higher rate of monomer elution compared to the bulk-fills, except FBF, which showed a high amount of UDMA release.

## 1. Introduction

The evolution led to contemporary resin-based composites (RBCs) showing high clinical success and survival rates [1,2,3,4]. However, despite the continuous development of RBCs, there are some shortcomings, such as polymerization volume shrinkage, incomplete degree of conversion (DC) of the matrix monomers and their release into the oral cavity and pulp space. The degree of conversion of an RBC is an important factor in determining the mechanical properties of the material and its biocompatibility [5,6]. A lower conversion rate will influence the physical performance of the RBC, and increased elution of monomers have been reported [7,8]. However, the DC% for adequate clinical performance has not yet been determined; only a negative correlation of *in vivo* abrasive wear depth with DC has been established in the range of 55–65 DC% [9,10]. To prevent clinical failures and decrease the elution of unreacted monomers, some practical strategies are recommended, including alternative light curing protocols [11], the use of flowable cavity liners [12] and incremental filling techniques [13]. The generally-accepted maximal layer thickness that provides adequate light penetration and photo-polymerization is 2 mm [7,14,15,16]. However, restoring cavities with RBC increments of 2 mm in thickness is time consuming; voids may be included; and this implies a risk of contaminations between the increments [17]. Thus, bulk-fill RBCs were developed to avoid the aforementioned disadvantages [18]. Literature data suppose better light transmittance of these materials to allow for a reported depth of cure in excess of 4 mm [19,20,21]. According to the manufacturer’s recommendation, bulk-fill materials are indicated as basing materials or permanent restorative materials. *In vitro* studies showed that the application of one thicker increment of bulk-fill composite could be equally successful in marginal adaptation, cavity-bottom adhesion and in depth of cure as the conventional layering technique [20,22]. Bulk-fill materials showed lower shrinkage stress and exhibited an acceptable creep deformation and reduced cuspal deflection when compared to conventional RBCs [17,23,24]. In a recent investigation, mechanical properties, including degree of cure, were shown to be constant within the 4-mm increment [25]. In the dental literature, studies investigated the amount of eluted monomers; thus, the biocompatibility and clinical performance of bulk-fill flowable RBC base materials are limited [20,21,22,24,26]. Biocompatibility depends on the quality and quantity of released monomers and their derivates, which can irritate the pulp, the soft tissues of the oral cavity and may lead eventually to a toxic reaction [27,28]. Several factors, such as the DC, the specimen thickness, the chemical composition, the filler particle type and content, the porosity and the solvent can influence the amount of released monomers [29].

The aim of this study was to assess the DC and the amount of released BisGMA, TEGDMA and UDMA monomers of some low-viscosity bulk-fill composite materials in a 4-mm layer thickness compared to that of conventional flowable one in a 2-(positive control) and a 4-mm (negative control) thickness, using micro-Raman spectroscopy and HPLC.

## 2. Results

### 2.1. Degree of Conversion-Micro-Raman Spectroscopy

**Figure 1:**
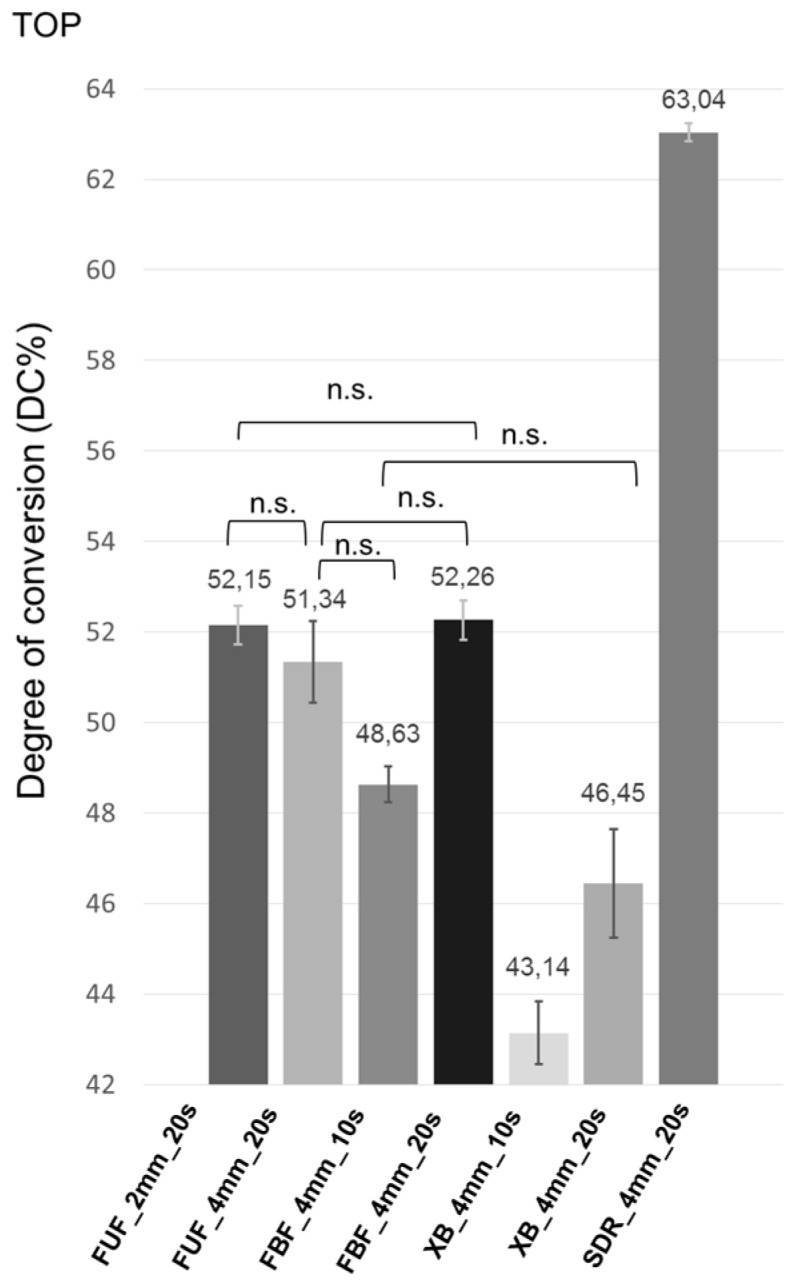
Mean DC% and 95% confidence intervals of the top surface of the samples (abbreviations: DC, degree of conversion; n.s., not significant difference; FUF_2mm_20s, Filtek Ultimate Flow in a 2-mm layer thickness cured for 20 s; FUF_4mm_20s, Filtek Ultimate Flow in a 4-mm layer thickness cured for 20 s; FBF_4mm_10s, 4 mm-thick Filtek Bulk Fill light cured for 10 s; FBF_4mm_20s, 4 mm-thick Filtek Bulk Fill light cured for 20 s; XB_4mm_10s, 4 mm-thick X-tra Base light cured for 10 s; XB_4mm_20s, 4 mm-thick X-tra Base light cured for 20 s; SDR_4mm_20s, SureFil SDR Flow in a 4-mm layer thickness cured for 20 s).

**Figure 2:**
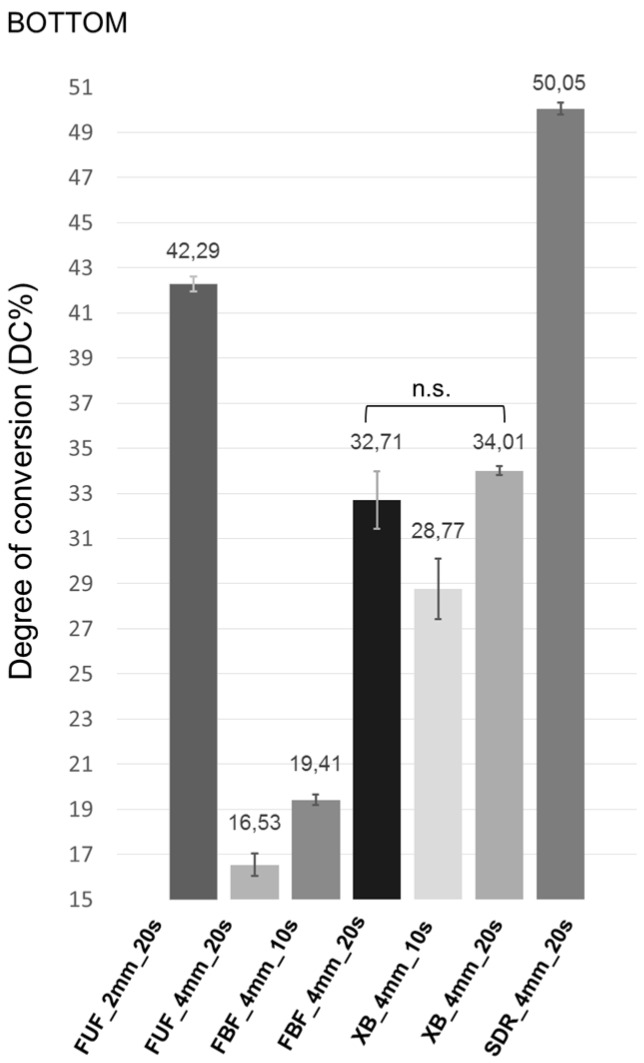
Mean DC% and 95% confidence intervals of the bottom surface of the samples (abbreviations: DC, degree of conversion; n.s., not significant difference; FUF_2mm_20s, Filtek Ultimate Flow in a 2-mm layer thickness cured for 20 s; FUF_4mm_20s, Filtek Ultimate Flow in a 4-mm layer thickness cured for 20 s; FBF_4mm_10s, 4 mm-thick Filtek Bulk Fill light cured for 10 s; FBF_4mm_20s, 4 mm-thick Filtek Bulk Fill light cured for 20 s; XB_4mm_10s, 4 mm-thick X-tra Base light cured for 10 s; XB_4mm_20s, 4 mm-thick X-tra Base light cured for 20 s; SDR_4mm_20s, SureFil SDR Flow in a 4-mm layer thickness cured for 20 s).

The top and bottom surface DC values of the materials are presented in Figure 1 and Figure 2.

The mean DC values on the top surface of the materials were in the following order: SDR_4mm_20s > FBF_4mm_20s > FUF_4mm_2mm > FUF_4mm_20s > FBF_4mm_10s > XB_4mm_20s > XB_4mm_10s; however, the order of the mean DC values on the bottom surface was significantly different from the top surface: SDR_4mm_20s > FUF_2mm_20s > XB_4mm_20s > FBF_4mm_20s > XB_4mm_10s > FBF_4mm_10s > FUF_4mm_20s. Dunnett’s *t*-test showed that all of the investigated materials had statistically significant differences in DC% at the bottom surface when compared to each other, except between FBF_4mm_20s and XB_4mm_20s (*p* = 0.221). On the contrary, between FUF_2mm_20s and FUF_4mm_20s (*p* = 0.306), FUF_2mm_20s and FBF_4mm_20s (*p* = 1.000), FUF_4mm_20s and FBF_4mm_20s (*p* = 0.241), FUF_4mm_20s and FBF_4mm_10s (*p* = 0.059) and between XB_4mm_20s and FBF_4mm_10s (*p* = 0.063), Dunnett’s *t*-test did not show significant difference in DC% at the top surface. The conventional flowable composite FUF_4mm_20s had the lowest DC value at the bottom (16.53%), while SDR_4mm_20s (50.05%) had the highest DC value not only at the bottom, but at the top surface, as well. The extended curing time of FBF and XB from 10–20 s significantly increased the DC%, especially at the bottom surface.

### 2.2. Monomer Elution: HPLC

**Figure 3:**
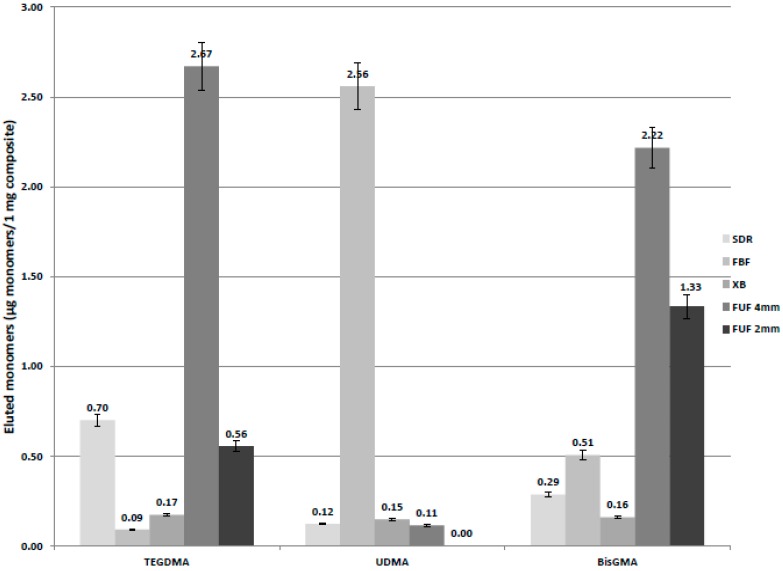
Amount of eluted monomers from bulk-fill and conventional flowable composites. (Abbreviations: SDR, SureFil SDR Flow in a 4-mm layer thickness light cured for 20 s; FBF, Filtek Bulk Fill in a 4-mm layer thickness light cured for 10 s; XB, X-tra Base in a 4-mm layer thickness light cured for 10 s; FUF 4 mm, Filtek Ultimate Flow in a 4-mm layer thickness light cured for 20 s; FUF 2 mm, Filtek Ultimate Flow in a 2-mm layer thickness light cured for 20 s).

Figure 3 shows the amount of eluted BisGMA, UDMA and TEGDMA from the 2 mm- and 4 mm-thick conventional flowable and the bulk-fill flowable RBC materials.

In spite of the fact that there is no BisGMA in SDR and XB according to the manufacturer’s information, there was a detectable amount of this monomer from these bulk-fill materials, such as TEGDMA from XB. In comparison, with Dunnett’s *t*-test, there were significant differences between the groups. More than a five-times higher elution rate was found in the amount of released BisGMA in the case of the 4 mm-thick conventional flowable FUF when this material was compared to the bulk-fill materials, and the difference in BisGMA release between the 2 mm- and 4 mm-thick FUF was almost twice (*p* < 0.001). The leached BisGMA from bulk-fill RBCs showed the following order: FBF > SDR > XB; the differences were statistically significant (*p* < 0.001). The FUF_4mm_20s had a statistically-significant higher rate of TEGDMA elution, as well, compared to the other materials (*p* < 0.001). Among bulk-fills, SDR showed a seven-times higher amount of TEGDMA elution. More than a twenty-times higher amount of eluted UDMA was observed in the case of FBF compared to SDR, XB and FUF; meanwhile, UDMA was not listed in the FUF’s technical product profile.

In the case of FUF and FBF, a high intensity peak was detected on the HPLC chromatogram at a different retention time than that of the above-mentioned three monomers, which was not identified and quantified in the lack of a standard monomer. However, according to the manufacturer’s information, it is probably the peak of procrylate monomer.

## 3. Discussion

In this study, the DC and the elution of unreacted monomers of different bulk fill and commercial flowable dental composites were assessed using micro-Raman spectroscopy and HPLC.

The setting process has a major influence on the mechanical and biological properties of RBCs [30]. Resin polymerization depends on the chemical structure of the monomer, filler characteristics, the photoinitiator concentration and the polymerization conditions [31]. Since polymerization conditions, such as layer thickness, intensity of the curing unit and exposure time, were standardized in this study, differences in the DC value of conventional and bulk-fill RBCs can be attributed to the different composition of the materials, mostly to variations in the chemistry of their resin matrix and the filler loading. In general, the manufacturers of bulk-fill RBCs were able to improve polymerization depth by the use of potent photoinitiator systems along with an increased translucency [18,32]. As light transmission is strongly dependent on material opacity [33], the observed higher DC% at a 4-mm specimen thickness for the investigated bulk-fills compared to the 4 mm-thick conventional flowable RBC might be a result of their reduced opacity. However, when the DC% of bulk fills was compared to the 2 mm-thick conventional flow, only SDR_4mm_20s produced a higher conversion rate. Higher translucency can also be achieved by the reduction in filler content [34]. It has been demonstrated by Halvorson *et al.* that increasing the filler-matrix ratio progressively decreases conversion, because an increased amount of filler particles is an obstacle for polymeric chain propagation [35]. According to Nomoto and Hirasawa [36], the depth of cure and, thus, the DC are affected by the filler’s light permeability, the monomer composition, the type and concentration of the initiator and the inhibitor/accelerator systems in the RBCs. In the present study, a significantly higher DC value was observed with the SDR_4mm_20s, and a tendency for a higher DC value was detected in FBF in comparison to XB cured for both 10 and 20 s. This finding is supported by other investigators [19,37,38,39] and might be explained by the higher filler content of XB, which may increase light scattering, causing a concurrent decrease of translucency for blue light [18].

Due to the presence of a photo-active modulator in the matrix system and the increased translucency, the manufacturer’s recommendation for the exposure time is 10 s for the universal shades, with the intensity of the curing unit ranging from 550–1000 mW·cm^−2^, while 20 s for SDR and FUF irrespective of the output intensity of the curing unit. Based on this fact, 5.5–10 J·cm^2^ of delivered energy should be enough for the adequate polymerization of bulk-fills. For a conventional RBC, the recommendation of a 21-J·cm^2^ and a 24-J·cm^2^ energy density has been made for the satisfactory conversion of a 2 mm-thick composite specimen [40]. Thus, as was expected, a 20-J·cm^2^ energy density was not enough for the acceptable polymerization rate of conventional flowable restorative samples in a 4-mm layer thickness; meanwhile, the decrease of layer thickness to 2 mm increased the polymerization rate with 61.5%. In the case of bulk fill XB and FBF, the extended curing time resulted in a 15.5% and a 40.7% increase in the rate of polymerization. In contrast with our findings, Finan *et al.* [19] observed higher DC% for SDR (59%) and XB (48%) using a quartz tungsten halogen (QTH) light curing unit (LCU) for polymerization, which operates at an output intensity of 650 mW/cm^2^ for 20 s; while Zorzin *et al.* [41] measured a 52% DC value for SDR, 63% for XB, 66% for FBF and 66% for FUF at a 4-mm layer thickness cured with an LED unit (1200 mW/cm^2^) for 20 s in the case of A2 shades. The possible explanation for this difference may be that wider samples in diameter were used to allow a higher degree of light penetration for polymerization or that Fourier transform infrared spectrophotometer was used to analyze the DC of RBC samples. However, Zorzin *et al.* concluded that extended curing time (30 s) had a positive effect on polymerization properties, so enhanced light curing of bulk-fills in deep cavities is recommended [41]. Similarly to our study, Li *et al.* [39] used micro-Raman spectroscopy to map the DC along a cross-section of bulk-fill and a conventional composite block. They measured 80% mean maximum DC for FBF and 77.3% for SDR; however, their study design was different from our design, as the investigators tested the curing profile of a thicker (16 mm× 6 mm× 12 mm) rectangular bulk-fill RBC block.

Besides the filler-matrix ratio, the DC is affected by the viscosity and reactivity of the polymerizable monomer, as well [42]. The DC of different monomer systems increases in the following order: BisGMA < BisEMA < UDMA < TEGDMA [43]. BisGMA is considered the most viscous monomer due to the strong intramolecular hydrogen bonding, which can decrease the reactivity and mobility of the monomer during the polymerization process. This might be one of the explanations for the significantly lower DC of the conventional flowable FUF_4mm_20s than that of other materials, as FUF contains the highest amount of BisGMA monomer in the resin matrix. In our study, the investigated bulk-fill RBCs are UDMA-based materials in combination with different types of monomers. Sideridou *et al.* found that UDMA, combining relatively high molecular weight with a high concentration of double bonds and low viscosity, was shown to reach higher final DC% values than BisGMA [43]. Low viscosity has a high impact on free radical migration. It was proven that the DC of RBC monomers is strongly influenced by the nature of the polymerizing monomers, for example more flexible monomers increase the rate of conversion [44]. Although the viscosity of UDMA is much lower than that of BisGMA, when it is mixed with the high molecular weight BisEMA or EBPADMA, it can significantly restrict the mobility of UDMA monomers and decrease their reactivity and conversion value [37,45]. This may explain the significantly lower DC value of FBF and XB cured for both 10 and 20 s than that of SDR_4mm_20s, as was reported by Alshali *et al.* [37]. SDR is similarly an UDMA/EBPADMA-based bulk-fill flowable composite; however, it contains TEGDMA, which has a synergistic effect on the rate of polymerization, and thus, the DC value of this monomer is significantly higher than that of the other investigated bulk-fill materials.

In addition, according to the manufacturer’s technical information, a photo-active modulator in SDR may cooperate with camphorquinone (CQ), thereby facilitating polymerization.

In the present study, monomer elution from RBC was quantified using HPLC. This is a standard method used for the determination of monomer elution from RBCs [46]. The release of components has a potential effect on the structural stability and wear rate, as well as the biocompatibility of the material. The analysis of the elution of selected unreacted BisGMA, TEGDMA and UDMA will not provide an absolute measure of the quality of released components; thus, it is a limitation of this study.

Several factors may influence the monomer elution, such as the rate of polymerization, the chemical features of the solvent and the chemical nature of the leached components [5,47]. In the present study, 75% ethanol was used to extract most of the examined unreacted monomers from the polymerized composite samples in order to identify monomer quantity. The elution pattern of unreacted monomers is higher in ethanol than in water storage medium, because of their hydrophobic character, which can significantly reduce and rationalize examination periods. Water storage may simulate oral conditions better than ethanol; however, changeful oral parameters (pH, temperature, enzyme activities) are hardly simulated in water medium. According to our results there was a strong correlation between DC% and the amount of eluted monomers in the case of the conventional flowable RBC (FUF). This is a BisGMA/TEGDMA/procrylate-based material, and the highest amount of leached TEGDMA and BisGMA was observed with the lowest DC%. Meanwhile, the reduced layer thickness decreased the amount of released TEGDMA and BisGMA by five- and two-times, respectively. The possible reasons for the different values for these monomers could be the chemical nature, the different molecular weight and the reactivity of the molecules. Tanaka *et al*. [48] found that TEGDMA has higher mobility caused by its low molecular weight, resulting in a higher and faster rate of elution than the larger BisGMA and UDMA [47,48]. As a TEGDMA/UDMA-based material, SDR showed a high amount of TEGDMA release following FUF_4mm_20s. Similar to our findings, Cebe *et al.* also found a higher amount of eluted TEGDMA monomers than from the other bulk-fills, and the cumulative amount of eluted TEGDMA increased with time [49]. In their study, Łagocka *et al.* detected lower (8.4 µg/g) TEGDMA elution from SDR during the first 24 h of storage in 75% ethanol solvent; however, the elution rate rapidly decreased with time [50]. The reason for the increased elution of the high molecular weight BisGMA could be the low rate of polymerization explained by the hampered light penetration and the decreased photoinitiator activation in the conventional flowable composite at a 4-mm layer thickness. Among bulk-fill composites, FBF showed the highest rate of released UDMA and BisGMA. Cebe *et al.* also detected a higher rate of eluted BisGMA from FBF, especially at the 30-day time interval [49].

Considering the filler content, FBF has the lowest filler value among the investigated bulk-fill materials, which may influence the release of unreacted monomers. Comparing the molecular weight of BisGMA and UDMA, BisGMA has a higher weight; thus, there is quicker and more substantial UDMA release in a certain time interval. Among bulk fills, XB showed a significantly lower rate of eluted monomers. The lower solubility might be based on the higher (75%) filler content in contrast with the other bulk-fills. There are reports that illustrate a lower absorption rate in composite materials with high filler contents compared to materials with lower filler content [51,52,53]. The results of the present study, which are in line with these reports, showed a lower monomer elution rate from XB with higher filler content compared to the other investigated bulk-fills. The possible explanation might be the lower solvent absorption in XB samples, which resulted in less leachable component elution. This leads to the conclusion that the elution mechanism is complex and cannot be explained only by the degree of conversion. On the other hand, Sideridou *et al.* concluded that higher silane content has a positive effect on interfacial adhesion between filler and matrix [54]. As the filler particles are chemically bonded to the matrix monomers and oligomers by the silane coupling agent, their higher volume fraction can provide a more stable ligation for the unreacted, leachable monomers, decreasing their release into the solvent. The structure of the silane coupling agent and its bonding to the filler particle has a high impact on the solubility of the RBC [55].

The detected BisGMA from the XB samples might be impurities of the monomer matrix complex.

Considering the BisEMA content in the investigated bulk-fill materials, there was no information from the degree of ethoxylation of the bisphenol A molecule; therefore, it was not possible to identify in the lack of standard monomer. Compared to other composite resin monomers, BisEMA is not a single monomer molecule, rather belonging to a large series of ethoxylated bisphenol A-based dimethacrylates with an ethoxylation reaction of a very reactive ethylene oxide [56,57]. Therefore, the ethoxylation reaction is unselective and difficult to control, leading to different ethoxylated products and byproducts, which must be separated analytically [58].

## 4. Materials and Methods

BisGMA (98%), UDMA (≥97%) and TEGDMA (95%) (Sigma-Aldrich, Steinheim, Germany) were used as standard materials for the identification of the monomer peaks in the chromatograms. Filtek Ultimate Flow flowable nanocomposite samples were prepared as references. The investigated materials were the following: SureFil SDR Flow, X-tra Base and Filtek Bulk Fill. Table 1 shows the composition of the materials. All samples were stored in a 75% ethanol/water solution (Spektrum-3D, Debrecen, Hungary). Acetonitrile (ACN) (VWR International, Leuven, Belgium) was used for the preparation of the mobile phase for the HPLC separation.

### 4.1. Preparation of the Composite Resin Specimens

The flowable bulk-fill RBCs (SDR, XB, FBF) were poured into a stainless steel mold with a size of 3 mm in diameter × 4 mm in thickness (*n* = 3 × 5) and positioned on a glass slide. As the negative control, conventional flowable composite (FUF) samples were used also with a size of 3 mm in diameter × 4 mm in thickness (*n* = 5) to be comparable with bulk-fill RBCs and with a size of 3 mm in diameter × 2 mm in thickness (*n* = 5) as a positive control. During preparation, each sample was measured to obtain samples of similar weight and volume. The top and the bottom of the RBC were covered with a polyester (Mylar, Dentamerica Inc., San Jose Ave, CA, USA) strip in order to avoid contact with oxygen, which is an inhibitor of the polymerization. The specimens were irradiated with a light-emitting diode (LED) curing unit (λ = 420–480 nm; LED.C, Woodpecker, Guilin, China) with the recommended exposure time at a light intensity of 1100 mW·cm^−2^ with an irradiated diameter of 10 mm. The manufacturer’s instruction for curing time at a 4-mm thickness and universal shades is 10 s in FBF and XB bulk-fill RBCs. In the case of these two materials, the effect of extended curing time (20 s) was also investigated. For SDR, the recommended exposure time is 20 s without giving a suggested value for the light intensity. In the case of A2 shade conventional flowable RBC, a 20-s exposure time at a 2-mm thickness is recommended; however, to investigate and to compare the DC value and the amount of released monomers, this product was also used in a 4-mm thickness and was irradiated only for 20 s. Table 2 summarizes the abbreviations of the prepared samples.

A radiometer (SDS, Kerr, Danbury, CT, USA) was used to control the intensity of the curing unit before and after the light exposition. The tip of the curing light guide was positioned parallel and 1 mm above the composite sample. One day after polymerization, the specimens were measured with micro-Raman spectroscopy. For the dissolution of the unreacted monomers the specimens were stored in 1 mL of 75% ethanol/water solution for 72 h in darkness at room temperature. After 3 days, the amount of dissolved unpolymerized monomers was analyzed with reverse-phase high-performance liquid chromatography (RP-HPLC) from the ethanol solutions.

### 4.2. Micro-Raman Spectroscopy Measurement

The polymerized composite samples were examined using a Labram HR 800 Confocal Raman spectrometer (HORIBA JobinYvon S.A.S., Longjumeau Cedex, France) 24 h after polymerization. During the micro-Raman measurements, a 20-mW He-Ne laser with a 632.817-nm wavelength was applied; the spatial resolution was ~1.5 µm; the spectral resolution was ~2.5 cm^−1^; with magnification of 100× (Olympus UK Ltd., London, UK), applying a D 0.3 filter (~1.98 mW on the sample). The spectra were taken on the top and also on the bottom surface of the composite specimens at three random locations. The integration time was 10 s, and ten acquisitions were averaged for each geometrical point. Uncured composite spectra were measured as a reference. These samples were placed between two non-fluorescent glass slides. Post-processing of spectra was performed using the dedicated software LabSpec 5.0 (HORIBA JobinYvon S.A.S., Longjumeau Cedex, France), and the Levenberg–Marquardt non-linear peak fitting method was applied for the best fit [59,60]. The following equation was used to calculate the ratio of the double-bond content of monomer to polymer in the composite:

(1) DC%=(1−(RcuredRuncured))×100

where *R* is the ratio of aromatic and aliphatic C=C bonds at peak intensities of 1639 cm^−1^ and 1609 cm^−1^ in cured and uncured composite samples, respectively [43,61].

### 4.3. RP-HPLC Measurements

The RP-UHPLC system (Dionex Ultimate 3000, Thermo Fisher Scientific Inc, Sunnyvale, CA, USA) consists of a Dionex LPG 3400 SD gradient pump, Dionex ACC 3000 autosampler and a Dionex UWD 3400 RS UV–VIS detector (Dionex GmbH, Germering, Germany). Data acquisition was completed using Chromeleon software integrated in Hystar (version: 3.2). The separations were performed on a Synergi HYDRO-RP (particle size: 4 μm; pore size: 8) (Phenomenex, Gen-Lab, Budapest, Hungary) column (150 mm × 2.00 mm) with gradient elution. The composition of Eluent “A” was 40% *v*/*v* ACN in bidistilled water, whereas Mobile Phase “B” was composed of 95% *v*/*v* ACN and 5% bidistilled water. During the 30-min chromatographic separation, the “B” eluent content increased from 20%–100%. The flow rate was 0.3 mL·min^−1^. As the regeneration of the stationary phase, Mobile Phase B content was decreased from 100% down to 20% in 1 min, and after 31–46 min, the system was washed with 20% “A”.

The detection of the eluted monomers was at the following wavelengths: 205, 215, 227 and 254 nm. Two hundred five nanometers was found to be optimal; therefore, the evaluation relied on the data collected at this wavelength. Each separation was implemented at room temperature.

The amount of the eluted monomers was calculated by the calibration curve with the areas under the curve of peaks produced by the monomers, respectively. The TEGDMA, UDMA and BisGMA standard solutions had retention times of 2.95, 5.08 and 6.88 min, respectively, whereas the peaks were well separated from each other.

### 4.4. Validation of the Monomer Determination the Limit of Detection and the Limit of Quantification

The detection limit (determined as the amount of monomers giving a peak height 5-times higher than the noise level) of TEGDMA is 0.018 pmol (5.19 pg), UDMA, 0.015 pmol (7.196 pg), and BisGMA, 0.007 pmol (3.556 pg). The quantification limit of the method (the peak height of the monomers 10-times higher than the noise level) was low for TEGDMA is 0.036 pmol (10.382 pg), for UDMA, 0.031 pmol (14.392 pg), and for BisGMA, 0.014 pmol (7.1120 pg). Calibration was carried out in the range of 1–50.0 µg·mL^−1^ monomers, respectively. A calibration curve was plotted by the measurement of standard solutions at 205 nm (*R*^2^) 0.9924 for TEGDMA, 0.9966 for UDMA and 0.9996 for BisGMA, respectively. All injections were repeated three times.

### 4.5. Statistical Analysis

The statistical analysis was performed using SPSS (Statistical Package for Social Science, SPSS Inc., Chicago, IL, USA) software for Windows. The values for the degree of conversion and for residual monomers between the studied test groups were compared by a one-way analysis of variance (ANOVA) test followed by Dunnett’s *t*-test at the α = 0.05 level.

## 5. Conclusions

Within the limitation of the present study, the following can be concluded:

(1) Among the investigated low viscosity bulk fill and conventional flowable RBCs, SDR showed the highest DC value at the top and bottom surface of the samples.
(2) The DC values of the 4 mm-thick bulk-fill composites SDR, FBF, XB were significantly higher than that of the 4 mm-thick conventional composite (negative control) studied; meanwhile, only SDR bulk-fill resulted in a higher DC value compared to that of the 2 mm-thick conventional flowable RBC (positive control).
(3) Although the recommended exposure time by the manufacturers for the universal shade FBF and XB is 10 s (with a 1000-mW/cm^2^ curing unit), extended (20 s) curing time significantly increased the DC% value.
(4) The amount of released BisGMA and TEGDMA monomers from the bulk-fill composite materials was generally lower than from the conventional composite.
(5) Among bulk fills, in spite of the highest DC%, SDR showed the highest rate of TEGDMA elution; meanwhile, the highest amount of UDMA was eluted from FBF.

## Figures and Tables

**Table 1:** Materials, manufactures and composition.

Group	Material	Code	Manufacturer	Shade	Organic Matrix	Filler	Filler Loading	LOT Number
Bulk-fill composite	SureFil SDR Flow	SDR	Dentsply Caulk, Milford, DE, USA	U	Modified UDMA, EBPADMA, TEGDMA	Ba-Al-F-B silicate glass, Sr-Al-F silicate glass	68 wt %	1202174
	x-tra base	XB	Voco, Cuxhaven, Germany	U	UDMA, BisEMA	no information	75 wt %	1305261
	Filtek Bulk Fill	FBF	3M ESPE, St Paul, MN, USA	U	BisGMA, UDMA, BisEMA(6), TEGDMA, substituated dimethacrylate, Procrylat resin	silane treated zirconia/silica, ytterbium trifluoride	64.5 wt %	N414680
Conventional flowable composite	Filtek Ultimate Flow	FUF	3M ESPE, St Paul, MN, USA	A2	BisGMA, TEGDMA, substituated dimethacrylate, Procrylat resin	silane treated zirconia/silica, ytterbium trifluoride	65 wt %	N652740

Abbreviations: U, universal; UDMA, urethane dimethacrylate; EBPADMA, ethoxylated Bisphenol A dimethacrylate; TEGDMA, triethylene glycol dimethacrylate; BisEMA, Bisphenol A polyethylene glycol diether dimethacrylate; BisGMA, Bisphenol A diglycidil ether dimethacrylate; Procrylate, reacted polycaprolactone polymer. Missing entries are not specified by the manufacturer.

**Table 2:** Sample preparation with layer thickness and exposure time.

Abbreviation	Material	Layer Thickness (mm)	Exposure Time (s)
FUF_2mm_20s	Filtek Ultimate Flow	2	20
FUF_4mm_20s	Filtek Ultimate Flow	4	20
FBF_4mm_10s	Filtek Bulk Fill	4	10
FBF_4mm_20s	Filtek Bulk Fill	4	20
XB_4mm_10s	X-tra Base	4	10
XB_4mm_20s	X-tra Base	4	20
SDR_4mm_20s	SureFil SDR Flow	4	20

## Abbreviations

DC	degree of conversion
HPLC	high performance liquid chromatography
RP-HPLC	reverse-phase high-performance liquid chromatography
SDR	SureFil SDR Flow
XB	X-tra Base
FBF	Filtek Bulk Fill
FUF	Filtek Ultimate Flow
FUF_2mm_20s	Filtek Ultimate Flow in a 2-mm layer thickness cured for 20 s
FUF_4mm_20s	Filtek Ultimate Flow in a 4-mm layer thickness cured for 20 s
FBF_4mm_10s	4 mm-thick Filtek Bulk Fill light cured for 10 s
FBF_4mm_20s	4 mm-thick Filtek Bulk Fill light cured for 20 s
XB_4mm_10s	4 mm-thick X-tra Base light cured for 10 s
XB_4mm_20s	4 mm-thick X-tra Base light cured for 20 s
SDR_4mm_20s	SureFil SDR Flow in a 4-mm layer thickness cured for 20 s
UDMA	urethane dimethacrylate
BisGMA	bisphenol A diglycidyl ether dimethacrylate
TEGDMA	triethylene glycol dimethacrylate
BisEMA	bisphenol A polyethylene glycol diether dimethacrylate
EBPADMA	ethoxylated bisphenol A dimethacrylate
RBC	resin-based composite
SD	standard deviation
QTH	quartz tungsten halogen
LCU	light curing unit
CQ	camphorquinone
CAN	acetonitrile
LED	light emitting diode
LOD	limit of detection
LOQ	limit of quantification
